# 

**Published:** 1889-06-08

**Authors:** 


SATURDAY, JUNE 8th, 1889.

**A Successful Record** - 143

**The Improvement of Idiots** - 144

**Hydrophobia** - 145

**Vital Statistics** - 146

**The Doctor's Pulpit.**—Man and his Enemies - 147

**The Causation of Disease** - 148

**Within the Wards.**—Dangers of Raw Milk—Tendon Grafting - 149

**Notes and News** - 150

**Everybody's Page** - 152

**Annotations.**—An Open-Air Academy—A Modern Cain—The Dangerous Season - 153

**The Hospitals Association.** — Dr. Bristowe's Address - 154

**"Her Heart's Mistake."** By E. D. Cumming, Author of "An Ordeal by Silence" - 156

**Amusements—Scraps and Gleanings** - 158

**EXTRA SUPPLEMENT—THE NURSING MIRROR.**

**En Passant.**—Practical Appreciation—Breakfast Baskets—Examination Questions—Nursing Lectures—Where Gifts Should Go?—The Care of the Insane—A Warning for Institutions - xxxv

**A First Experience** - xxxvi

**Nurses and Gossip** - xxxvi

**An Evening at the Grosvenor** - xxxvii

**Recompense** - xxxvii

**The Nurse's Bookshelf.**—Speaking Plainly - xxxvii

**Everybody's Opinion.** — Zenana Medical College — A Nurses' Club—The Nursing Craze - xxxviii

**Good Temper** - xxxviii

**Appointments.—Vacancies** - xxxviii

**Notes and Queries** - xxxviii

